# Investigation of kinetics, isotherms, thermodynamics and photocatalytic regeneration of exfoliated graphitic carbon nitride/zeolite as dye adsorbent

**DOI:** 10.1038/s41598-023-41262-7

**Published:** 2023-08-29

**Authors:** Hajar Farhadi, Narjes Keramati

**Affiliations:** Department of Nanotechnology, Faculty of New Sciences and Technologies, Semnan University, Semnan, Iran

**Keywords:** Chemistry, Engineering, Nanoscience and technology

## Abstract

A novel exfoliated graphitic carbon nitride and clinoptilolite nanocomposites (Ex.g-C_3_N_4_/CP and g-C_3_N_4_/CP with a various ratios of g-C_3_N_4_ to CP) were prepared by facile method. This study evaluates the adsorption of methylene blue (MB) on the surface of synthesized adsorbents. The as-prepared composites were characterized by XRD, FT-IR, FESEM, BET and DRS. Batch experiments were carried out under various conditions, such as the amount of adsorbent and solution pH. The optimum batch experimental conditions were found under the response surface methodology. The Ex.g-C_3_N_4_/CP presented maximum removal of MB as compared to others. The removal efficiency of the as-prepared nanocomposite was significantly elevated owing to the synergistic effects. The adsorption capacities of MB (10 ppm) on Ex.g-C_3_N_4_/CP was 54.3 mg/g. The adsorption process by both composites (g-C_3_N_4_/CP and Ex.g-C_3_N_4_/CP) showed well-fitting with the Elovich kinetic model, and Langmuir isotherm. The thermodynamic study suggested that the adsorption of MB was a spontaneous and endothermic process. The reusability of g-C_3_N_4_/CP1:2 and Ex. g-C_3_N_4_/CP in removing of MB (10 ppm, pH = 9) was studied by photocatalytic regeneration under visible irradiation for three consecutive cycles. The results obtained from the experimental analyses showed that the removal of MB was easy treatment, eco-friendly, and high yield.

## Introduction

Water pollution by industries such as textile and petrochemical, plant pesticides is one of the biggest problems in the world^1, 2^. One of the main sources of water pollution is dyes such as MB, a cationic dye which was widely used in textiles, leather, printing, paint, plastic, and medicine^3–5^. In recent years, various processes such as adsorption, ion exchange, sedimentation, reverse osmosis, filtration and oxidation have been used. Among them, adsorption is used due to its high efficiency, low cost, and simplicity^6^. More recently, the investigations have focused on the use of natural adsorbents such as activated carbon (AC) for dye adsorption^1, 4, 7–9^. Altintig et al., were investigated adsorption potential of AC for the removal of dye from aqueous solution. The sorbents had considerable high adsorption capacities as 103.64–106.54 mg/g at 298–318 K under optimized batch conditions, pH 6, mixing time 60 min and adsorbent dose 0.1 g/100 mL^7^. In other research, the AC derived from waste scrap tires was modified by Fe and Ce nanoparticles and then used as an adsorbent for RhB dye removal by Tuzen et al. The results showed that the developed magnetic AC/Fe/Ce nanocomposite had a relatively high adsorption capacity of 324.6 mg/g at pH 5^1^.

Graphitic carbon nitride (g-C_3_N_4_) is a polymer material that has a potential for adsorption, due to its low cost, environmental friendliness, high thermal and chemical stability^10^. The g-C_3_N_4_ is usually synthesized by thermal polymerization of urea, thiourea, cyanamide and melamine^11, 12^. The nanostructures of this material are suitable candidates for adsorbing pollutants. The g-C_3_N_4_ nanostructures can be easily obtained by exfoliation (by ultrasonic, thermal and chemical methods) of its bulk. The thermal method can be considered as a low cost, large scale and environmentally friendly method^3, 13, 14^. Despite the above advantages, g-C_3_N_4_ tends to agglomerate during the synthesis, which will lead to the reduction of surface active sites for pollutant adsorption^15^. The use of compounds with a high surface area as a base has been suggested^16, 17^. In 2016, Dunn and co-workers studied the activity of g-C_3_N_4_ based on MCM-41. The results of their research indicated that dispersion of g-C_3_N_4_ on the surface of the porous base had a positive effect on its adsorption capacity^17^. In another study, Wang et al., in 2019, deposited g-C_3_N_4_ on the surface of montmorillonite and investigated its performance in lead adsorption^18, 19^. Among the natural zeolites, Clinoptilolite (CP) is one of the best and most abundant types with the ability to be widely used^20^. Both Clinoptilolite zeolite with negative surface charge and g-C_3_N_4_ with -NH_2_, -NH-, = N- functional groups tend to adsorb cationic dyes.

In this study, for the first time, the novel graphitic carbon nitride/clinoptilolite composite (g-C_3_N_4_/CP) with synergistic effect of both components by different ratios of g-C_3_N_4_ were synthesized and investigated as MB adsorbent. Also, for comparison, a composite of exfoliated graphitic carbon nitride/clinoptilolite (Ex. g-C_3_N_4_/CP) was also synthesized. Here, we present a simple and large yield synthesis route for them through the pyrolysis of urea under ambient pressure. Urea is used as a precursor due to its low cost. The influence of different adsorption parameters, including adsorbent concentration and solution pH was investigated by response surface method (RSM). Also, adsorption isotherms, kinetics and thermodynamics were studied to evaluate and compare the adsorption performance of g-C_3_N_4_/CP and Ex.g-C_3_N_4_/CP. Graphitic carbon nitride not only helps CP in adsorbing MB, but due to its ability to be activated under visible light radiation, the possibility of photocatalytic regeneration of the composite will also be available, a significant feature for industrial applications. This fundamental study will be helpful to design a new adsorbent for the removal and photodegradation of MB dye from the aqueous solutions.

## Materials and methods

### Materials

Urea (C_6_H_11_NO_4_), methanol (CH_3_OH), sodium hydroxide (NaOH), nitric acid (HNO_3_), sodium chloride (NaCl) and MB dye were purchased from the Merck Company. All chemicals were analytically pure. Clinoptilolite as natural zeolite was provided from Negin Powder Semnan Company (Iran). Distilled water was used throughout the experiment.

### Preparation of adsorbents

**Figure 1:**
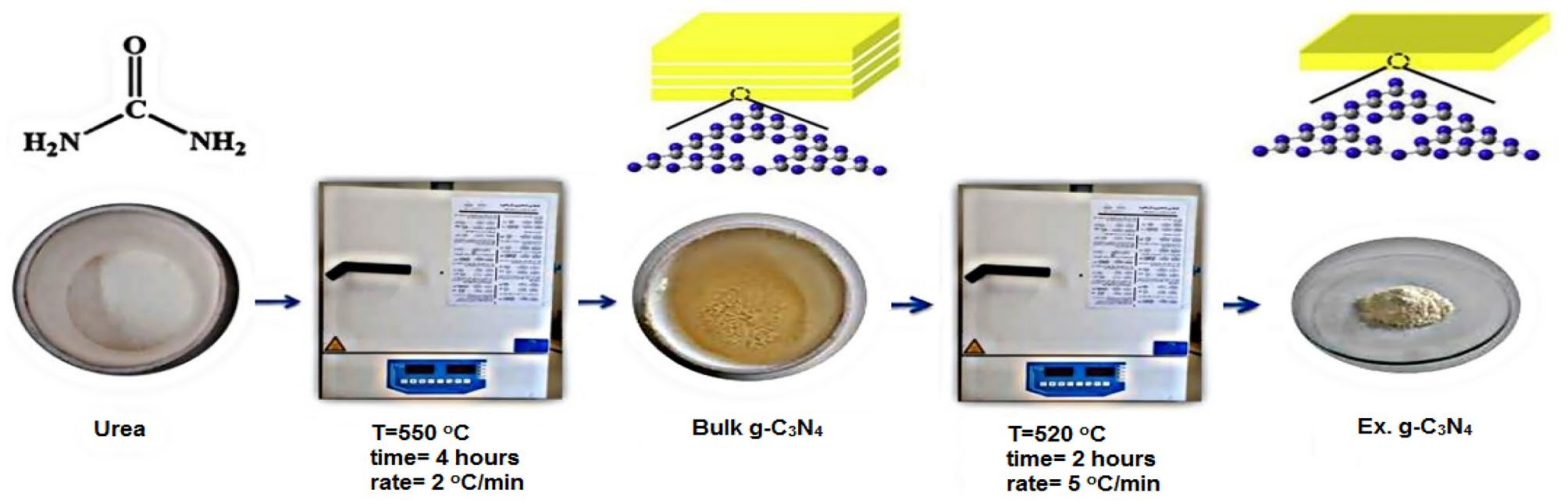
Steps for the synthesis of bulk g-C_3_N_4_ and Ex.g-C_3_N_4_ samples.

In order to remove natural zeolite impurities, it was washed with distilled water for one hour and then dried in an oven at 70 °C for one hour and named as CP. Bulk graphitic carbon nitride was synthesized by thermal polymerization. For synthesis, 16 g of urea was poured into a 100 ml aluminum crucible with a lid, then it was heated for 4 h at 500 °C (rate of 2 °C/min), and finally a yellow powder product was obtained. It was named as bulk g-C_3_N_4_. Also, exfoliated graphitic carbon nitride was synthesized by thermal oxidation. First, 0.4 g of synthesized bulk g-C_3_N_4_ was poured into a 100 ml alumina crucible, then it was heated for 2 h at 520 °C (5 °C/min) and finally a pale yellow powder was obtained. It was named as Ex.g-C_3_N_4_ (Fig. 1).

Different amounts of synthesized bulk g-C_3_N_4_ were dispersed in methanol (50 ml) and exposed to ultrasonic radiation for 30 min. Then, different amounts of CP were added and stirred for 24 h. At the end, it was dried in the oven at 70 °C for 24 h. The synthesis conditions of three samples are given in the Table 1.

**Table 1:** The synthesis conditions of the samples.

Composite name	Bulk g-C_3_N_4_ (g)	CP (g)
g-C_3_N_4_/CP 1:1	2.5	2.5
g-C_3_N_4_/CP 1:2	2.5	5
g-C_3_N_4_/CP 2:1	5	2.5

For fabrication of the nanocomposite, 0.62 g of synthesized Ex.g-C_3_N_4_ was dispersed in water (50 ml) and exposed to ultrasonic radiation for 30 min. Then, 5 g of CP was added and stirred for 24 h. Then the obtained sample was dried in an oven at 70 °C for 24 h. The synthesized sample was named as Ex.g-C_3_N_4_/CP.

### Characterization of adsorbents

Brunker model D8 X-ray diffractometer was used to check the crystal structure. Fourier transform infrared spectroscopy (FTIR) Magna-IR Nikolate 550 was used to identify organic compounds. Zeiss sigma 300-HV field emission scanning electron microscope was used to determine the average size of particles and morphology. Also, Philips X-ray energy diffraction spectrometer (EDS) model XL30 was used. The transmission electron microscope (TEM) image was obtained from a Phillips model EM208S device. Nitrogen adsorption/desorption was done using Belsorp mini x device. Shimadzu model UV3600Iplus Diffuse Reflectance/Transmission Spectrometer (DRS) was used to determine light absorption ability.

### Adsorption experiments

Adsorption experiments were performed in batch mode by adding a specific amount of adsorbent to the known volume of MB solution on magnetic stirrers at room temperature. HCl and NaOH (1 M) solutions were used to adjust the pH. Finally, the adsorbent was separated by centrifugation and the concentration of the filtrate was determined by UV–Vis spectrophotometer at 664 nm. Removal efficiency® and equilibrium adsorption capacity were calculated by Eqs. (1) and (2), respectively. Where, q_e_ is the equilibrium adsorption capacity (mg/g), C_i_ is the initial concentration of MB (mg/L), C_e_ is the concentration of MB at the equilibrium (mg/L), V is the volume of MB solution (L) and M is the mass of the adsorbent (g)^21^.

1 $$R = \frac{{C_{i} - C_{e} }}{{C_{i} }} \times 100$$

2 $$q_{e} = \frac{{\left( {C_{i} - C_{e} } \right)V}}{M}$$

In order to determine the isotherm models, different concentrations of MB (5, 10, 30, 50 mg/L) was prepared at pH equal to 9 (according to the optimal conditions) with an adsorbent concentration of g-C_3_N_4_/CP1:2 and EX.g-C_3_N_4_/CP equal to 0.33 and 0.2 g/L, respectively. The solutions were stirred on a magnetic stirrer at ambient temperature and after the desired time; the samples were separated and their adsorption rate was determined. The regression coefficient was used to show how well the regression equation fits the data. Adsorption kinetics were investigated using g-C_3_N_4_/CP 1:2 and Ex.g-C_3_N_4_/CP in optimal conditions specified according to RSM results. The heat of adsorption refers to the thermal effect during the adsorption process, and the magnitude of it can reflect the degree of adsorption. The Gibbs free energy change (∆*G*^*o*^), enthalpy change (∆*H*^*o*^) and entropy change (∆*S*^*o*^) were investigated to determine whether the adsorption was spontaneous and analyze the driving force of adsorption. The experiments were carried out at 25, 35, and 45 °C with an initial MB concentration of 10 mg/L. Thermodynamic parameters can be calculated from the following Eqs. (3)–(4). Where *R* (8.134 J·mol^−1^·k^−1^) is the gas constant; *T* (K) presents the Kelvin temperature; (*K*=*qe*/*Ce*) is the distribution coefficient.

3 $$LnK = \, \left( {\Delta S^{0} /R} \right) - \left( {\Delta H^{0} /RT} \right)$$

4 $$\Delta G^{0} = \, \Delta H^{0} - T\Delta S^{0}$$

5 $$\Delta G^{0} = - RTLnk$$

## Results and discussion

### Characterizations of the adsorbents

**Figure 2:**
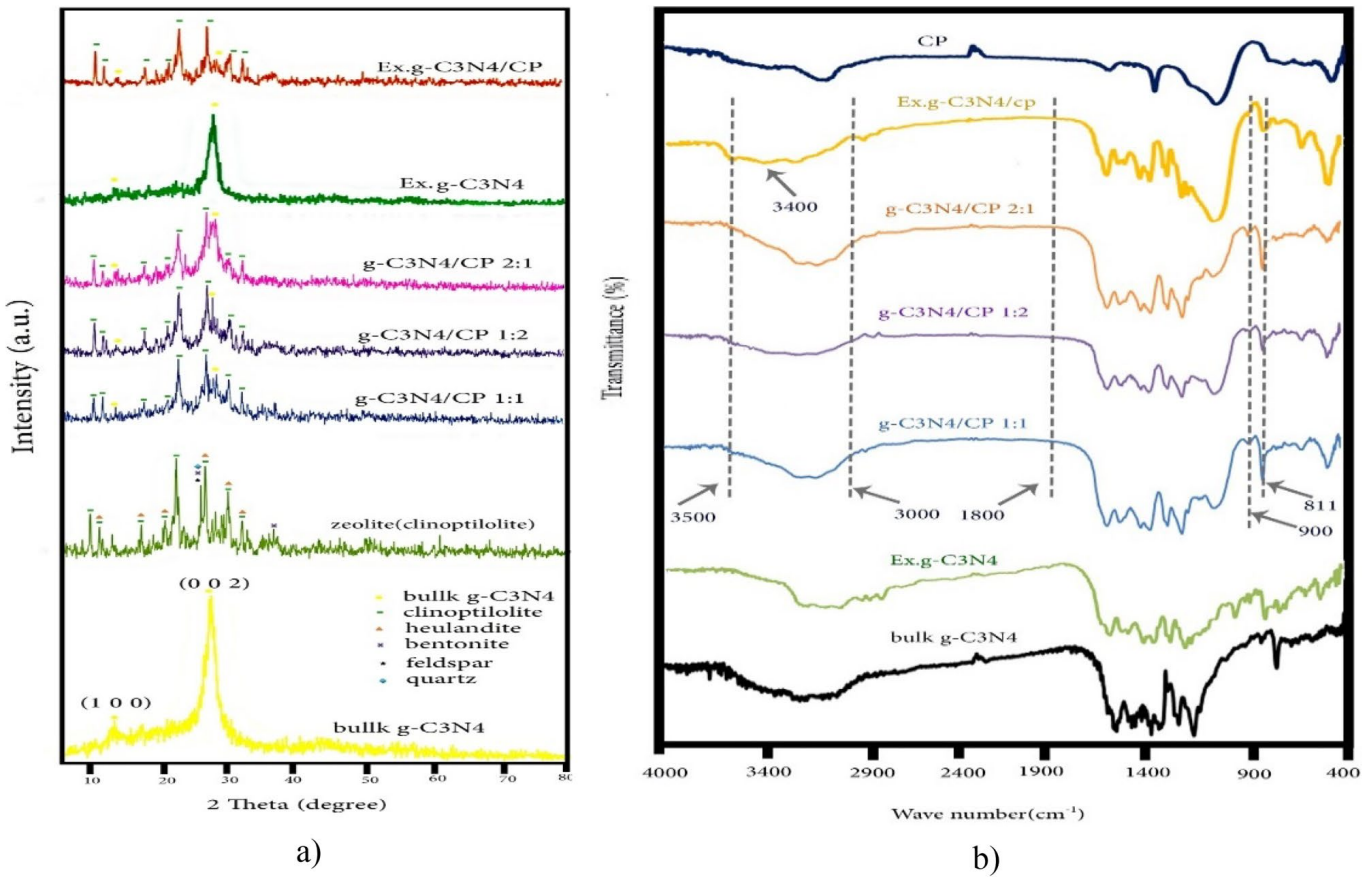
(**a**) XRD and (**b**) FT-IR of g-C_3_N_4_, CP, Ex.g-C_3_N_4_, g-C_3_N_4_/CP1:1, g-C_3_N_4_/CP1:2, g-C_3_N_4_/CP2:1 and Ex.g-C_3_N_4_/CP.

Crystal structure and phase purity of synthesized samples have been shown in Fig. 2a. The XRD pattern of natural zeolite show that it consists of significant amounts of Clinoptilolite, Hollandite, Biotite, Feldspar and Quartz. The characteristic XRD peaks of natural zeolite observed at 9.8, 11.24, 17.4, 20.8, 22.4, 26.7, 30 and 33°. This is well matched with the patterns of Clinoptilolite^22–24^. Besides, the 2θ values at 27.2 and 13° were corresponds to (002) and (100) planes of crystal faces of g-C_3_N_4_, that matched with the JCPDS card no (JCPDS No. 87–1526)^25^. These peaks have also appeared in the pattern of the Ex. g-C_3_N_4_. But after exfoliation, the intensity of the 27.2° was significantly reduced, and this indicated the successful synthesis^13^. Availability of both types of peaks in all composites confirms the successful incorporation of g-C_3_N_4_ into natural zeolite. Though, the characteristic peaks of natural zeolite was not shifted much, the stacking of g-C_3_N_4_ layers corresponds to the peak at 27.2°^26^ and in all composites has shifted to higher angles, which shows a reduction of the distance between graphitic carbon nitride layers^13^. The distance between layers in bulk g-C_3_N_4_, Ex.g-C_3_N_4_, g-C_3_N_4_/CP1:1, g-C_3_N_4_/CP1:2, g-C_3_N_4_/CP2:1 and Ex.g-C_3_N_4_/CP are equal to 0.324, 0.327, 0.316, 0.315, 0.317 and 0.323 nm. This can be explained that part of g-C_3_N_4_ inserted into the layer of zeolite. This is clearly indicated that the zeolite was well decorated on the g-C_3_N_4_. Also, the intensity of the peak at 27.2° gradually increases with the increase of g-C_3_N_4_, which indicates the successful integration of g-C_3_N_4_ with CP.

To further determine the composition information of g-C_3_N_4_, CP and all composites was further evidenced by FT-IR analysis (Fig. 2b). For bulk g-C_3_N_4_ and Ex.g-C_3_N_4_, three main regions can be seen. A sharp peak was observed at 811 and 1891 cm^−1^, which corresponds to 3-S triazine units. The characteristic peaks at 1231, 1327, 1417 and 1573 cm^−1^ were assigned to the N–C stretching vibrational state of aromatic rings, the peak at 1643 cm^−1^ was assigned to the N = C stretching vibrational state, and the peak at 2180 cm^−1^ was assigned to C≡N. Broad absorption peaks in the range of 3100 to 3300 cm^−1^ are related to the stretching vibrations of primary (NH_2_ groups) and secondary amines (NH = groups), and the peak near 3400 cm^−1^ is related to the stretching vibration of the interlayer H–OH bond^27, 28^. In the structure of Ex.g-C_3_N_4_, a peak at 3500 cm^−1^ was observed, which was attributed to –OH groups which are caused by the oxidation of g-C_3_N_4_ in air^29^. In the FT-IR spectra of Clinoptilolite, characteristic peaks in the range of 3750–2900 cm^−1^ are attributed to OH. Finally, the bending vibration of water is observed at 1627 cm^−1^. The sharp peak at 1013 cm^−1^ corresponds to Al atoms, the peaks observed in the range of 420–500 cm^−1^ are related to T–O^24, 30^. The FT-IR spectra of the composites can be clearly show that peaks from 900 to 1800 cm^−1^ are related to C–N H–C units. The broad peaks at 3500–3000 cm^−1^ are assigned to N–H and O–H stretching vibrations, which indicate the presence of NH or NH_2_ groups in them^30^. Also, the intensity of the peaks is weakened, which indicates that g-C_3_N_4_ with CP is not physically mixed together, but forms a lower energy structure^27^.

**Figure 3:**
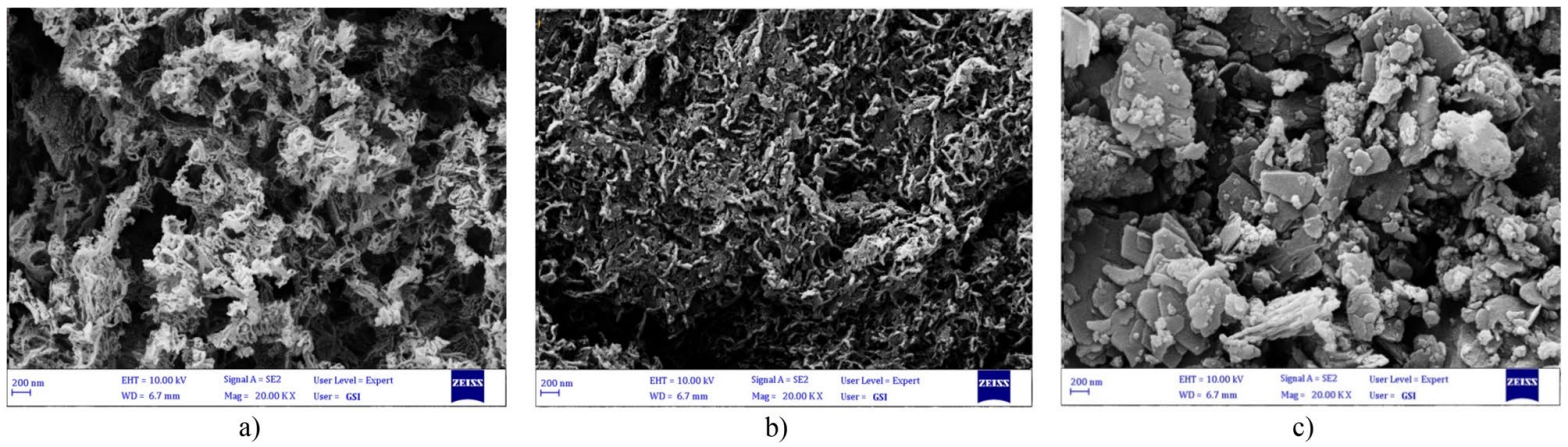
FESEM images of (**a**) bulk g-C_3_N_4_, (**b**) Ex. g-C_3_N_4_, (**c**) CP, (**d**) g-C_3_N_4_/CP1:2.

**Figure 4:**
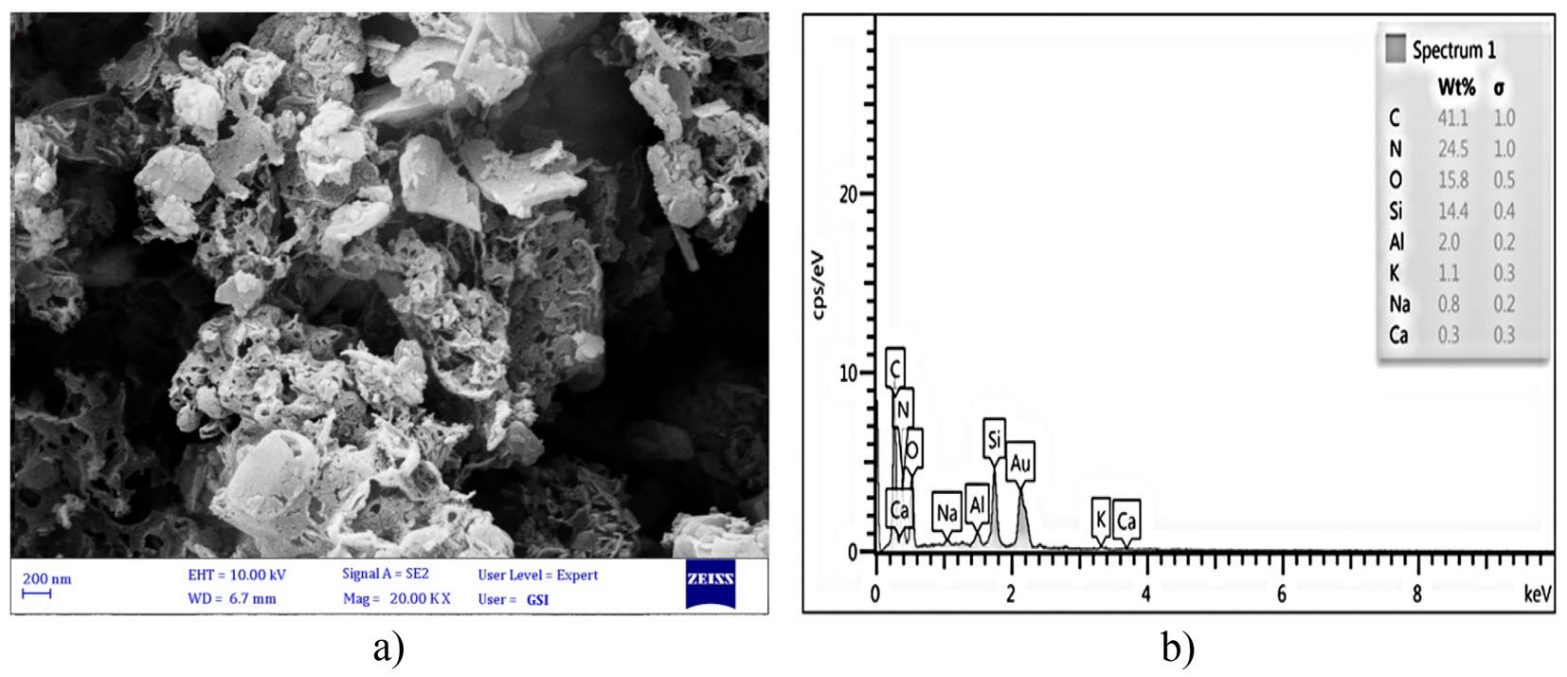
(**a**) FESEM images of g-C_3_N_4_/CP1:2, (**b**) EDS Spectra of g-C_3_N_4_/CP1:2.

The surface morphology of the bulk g-C_3_N_4_, Ex.g-C_3_N_4_ and CP was analyzed by SEM. Bulk g-C_3_N_4_ (Fig. 3a) has an irregular layered morphology similar to a honeycomb^15, 32^ and is formed in micrometer dimensions^31^. Ex.g-C_3_N_4_ (Fig. 3b)) has an irregular and layered morphology after thermal exfoliation^33^. It can be seen that CP (Fig. 3c)) has a flat and layered morphology. Also, flat particles are stacked together^34, 35^. According to Fig. 4a, both of g-C_3_N_4_ and CP are intertwined and block some mesoporous channels on the g-C_3_N_4_, which leads to a decrease in the surface area of the g-C_3_N_4_/CP1:2 composite compared to bulk g-C_3_N_4_. In other word, the surface and edges of the CP were coated uniformly and tightly with layer g-C_3_N_4_. The elemental composition of the as-synthesized composites was also confirmed by EDS (Fig. 4b). It was indicated that the synthesized compound was in high purity form.

**Figure 5:**
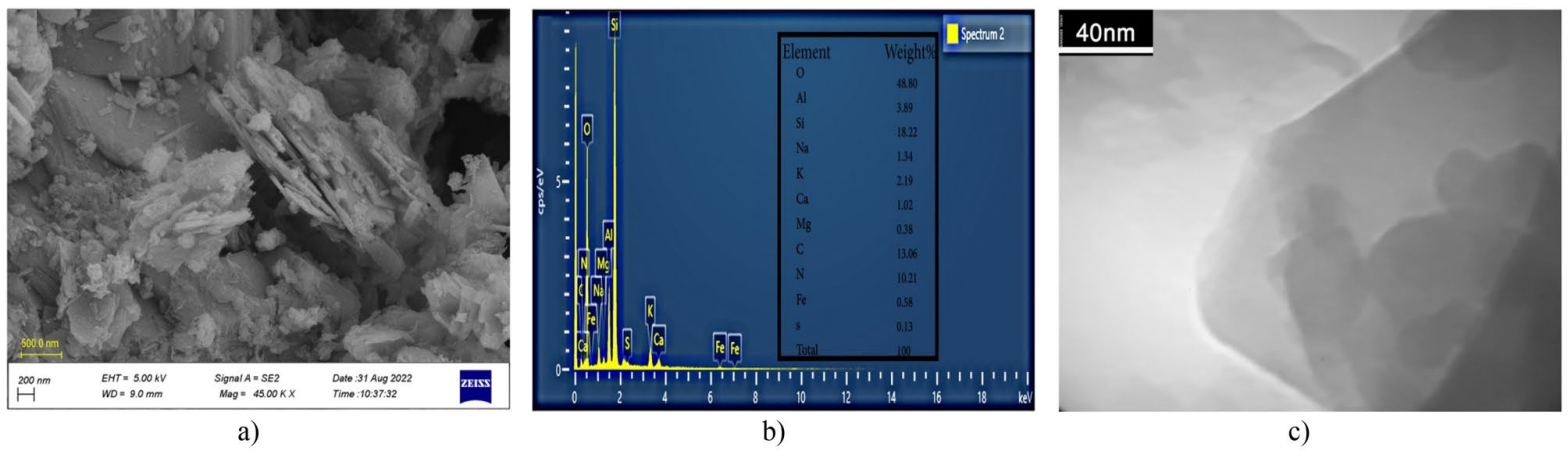
(**a**) FESEM images, (**b**) EDS Spectra, (**c**) TEM images of Ex. g-C_3_N_4_/CP.

According to Fig. 5, the Ex. g-C_3_N_4_ was appearing to be sheet like morphology. It is clearly confirmed that CP was randomly decorated on the g-C_3_N_4_ nano sheets.

**Figure 6:**
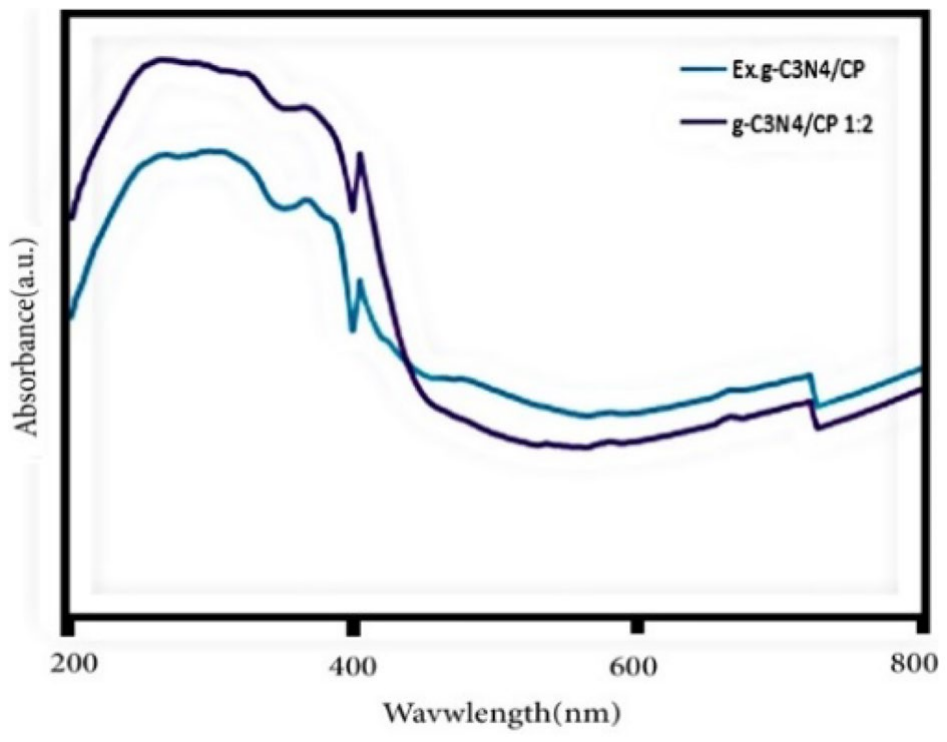
UV–vis spectra (DRS mode) of g-C_3_N_4_/CP1:2 and Ex.g-C_3_N_4_/CP composites.

The optical property of g-C_3_N_4_/CP1:2 and Ex.g-C_3_N_4_/CP composites during photocatalytic regeneration were investigated by UV–vis DRS (Fig. 6). Both of them are effective and great light harvesting efficiency for visible light. Therefore, both samples can be regenerated during the photocatalytic process under visible irradiation.

**Figure 7:**
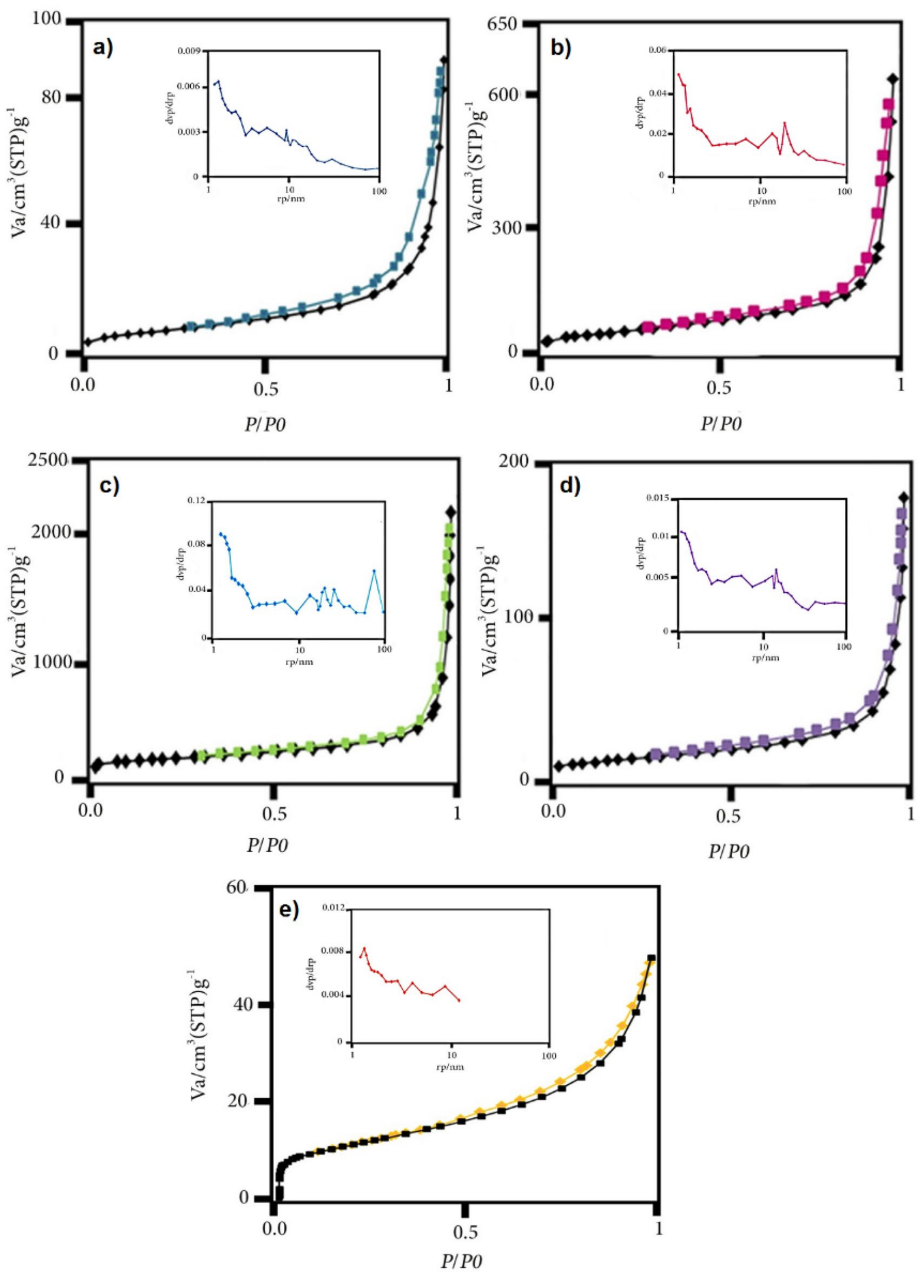
N_2_ adsorption/desorption isotherms of (**a**) CP, (**b**) bulk g-C_3_N_4_, (**c**) Ex. g-C_3_N_4_, (**d**) g-C_3_N_4_/CP1:2, (**e**) Ex. g-C_3_N_4_/CP and BJH (inside).

The surface area and pore size of samples were studied by N_2_ adsorption/desorption isotherm (Fig. 7, Table 2).

**Table 2:** The comparison of surface area, pore size and pore volume of synthesized samples.

Sample	Surface area [m^2^g^−1^]	Total pore volume [cm^3^g^−1^]	Mean pore diameter [nm]
CP	28.45	0.13	18.93
bulk g-C_3_N_4_	170.07	0.94	22.06
Ex.g-C_3_N_4_	336.39	3.04	36.18
g-C_3_N_4_/CP 1:2	42.45	0.27	25.08
Ex.g-C_3_N_4_/CP	38.10	0.07	7.64

According to the IUPAC classification, their isotherms are type IV and have a type of H3 hysteresis loop and show mesoporous materials. Surface area of bulk g-C_3_N_4_ is equal to 170.07 m^2^g^−1^, which by peeling, it has increased to 336.39 m^2^g^−1^^22, 36, 37^. The g-C_3_N_4_ was having lesser surface area than the composite, so the composite gave better adsorption properties than pristine g-C_3_N_4_. The synthesized samples have an average pore diameter between 2 and 50 nm, which indicates mesoporous materials. The BJH plot confirms the presence of mesoporous, which can be beneficial for the excellent adsorption performance.

### Adsorption of MB by synthesized samples

**Figure 8:**
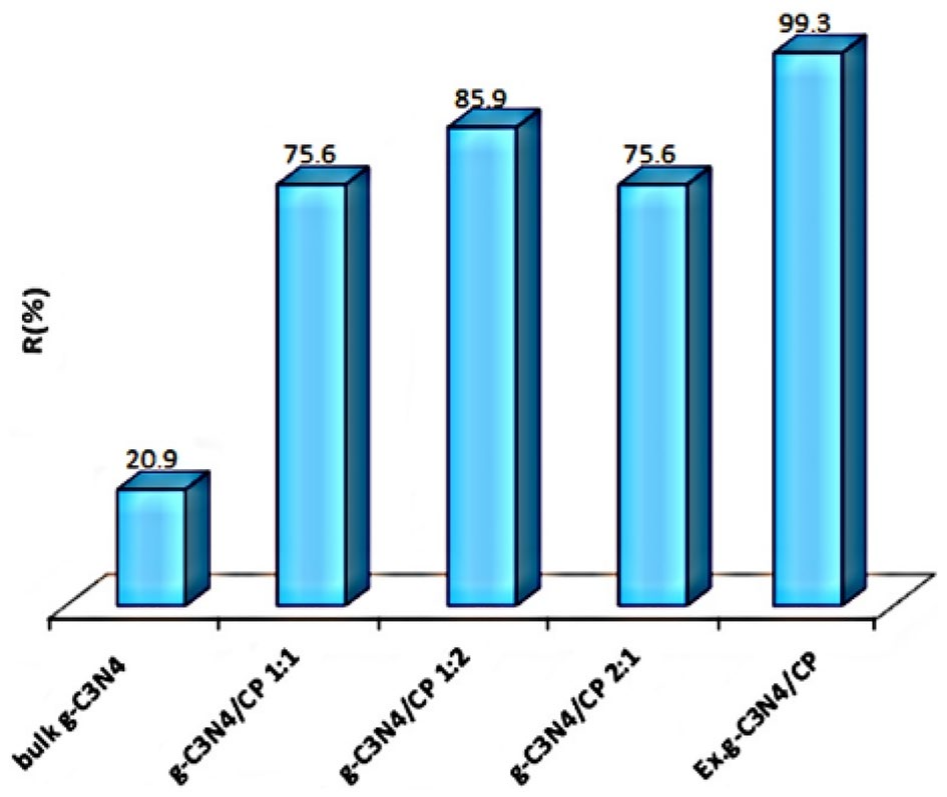
Adsorption efficiency of MB by synthesized samples (MB: 5 ppm, Adsorbent: 0.3 g/L, pH = 7, time 120 min).

Comparison of performance of synthesized samples in adsorption of MB was investigated (Fig. 8). Results indicated that all composites showed an improved performance compared to bulk g-C_3_N_4_. Also, the g-C_3_N_4_/CP 1:2 has the higher adsorption efficiency than g-C_3_N_4_/CP 1:1, g-C_3_N_4_/CP 2:1. And, the Ex.g-C_3_N_4_/CP nanocomposite has the highest adsorption efficiency. Therefore, by combining CP and g-C_3_N_4_, a synergistic effect was achieved in the composite in such a way that an adsorbent with high adsorption property along with the ability to regenerate under visible light radiation has been synthesized. In the continuation of the present study, the g-C_3_N_4_/CP1:2 and Ex.g-C_3_N_4_/CP samples were investigated and compared as the best synthesis samples and optimization of adsorption was done by using them.

With the aim of investigating the effective factors on the process, the interactions between the effective factors and their optimization, an experimental design based on response surface methodology (RSM) was carried out. Since the Box Behnken Design (BBD) set a mid-level between the original low and high level of the factors, avoiding the extreme axial points as in the central compound design (CCD), the latter was applied. Besides, BBD cannot estimate the full quadratic model for less than four factors^38, 39^. Two important operational parameters of the adsorption process (adsorbent concentration and initial pH value of the solution) were optimized by Design Expert 11 software. The removal efficiency of MB was selected as the response. The results of the design are presented in Table 3.

**Table 3:** The design matrix of the central complex; Factors, levels, experimental Responses (MB: 10 ppm, time: 180 min).

No	A: pH	B: Adsorbent concentration (g/L)	R (%)	
			g-C_3_N_4_/CP 1:2	Ex.g-C_3_N_4_/CP
1	9	0.2	65.4	96.6
2	6	0.3	62.8	96.2
3	3	0.4	19.7	97.3
4	10.3	0.3	95.5	95.8
5	6	0.44	26.8	96.8
6	1.8	0.3	48.7	95.2
7	6	0.3	62.8	96.2
8	6	0.16	13.4	94.7
9	6	0.3	62.8	96.2
10	9	0.4	82.7	96.6
11	6	0.3	62.8	96.2
12	3	0.2	22.4	93.1
13	6	0.3	62.8	96.2

The obtained data was in good agreement with the reduced quadratic model. The obtained models for predicting the removal percentage in the coded form for g-C_3_N_4_/CP1:2 and Ex.g-C_3_N_4_/CP composites are as Eqs. 6 and 7, respectively. The adequacy of the obtained model was checked with analysis of variance (Table 4). The regression coefficient of the models for both composites showed the adequacy and importance of the models.

6 $$R \, \left( \% \right) \, = - 131.66522 - 4.50895A + 1201.18808B + 16.66667AB + 0.55694A^{2} - 2098.75000B^{2}$$

7 $$R \, \left( \% \right) \, = \, 18.3309 + 1.99125A + 50.23896B - 4.68744AB - 0.041031A^{2} - 24.84001B^{2}$$

**Table 4:** ANOVA analysis.

Parameter	Sum of square		df		*F* value		*p* value	
	Ex.g-C_3_N_4_/CP	g-C_3_N_4_/CP 1:2	Ex.g-C_3_N_4_/CP	g-C_3_N_4_/CP 1:2	Ex.g-C_3_N_4_/CP	g-C_3_N_4_/CP 1:2	Ex.g-C_3_N_4_/CP	g-C_3_N_4_/CP 1:2
Model	7435.9	11.92	5	5	50.85	227.15	<0.0001	<0.0001
A	3705.97	0.4613	1	1	126.72	43.94	<0.0001	0.0012
B	140.70	1.93	1	1	4.81	184.07	0.0644	<0.0001
AB	100	100	1	1	3.42	448.57	0.1069	>0.0001
A^2^	174.78	4.71	1	1	5.98	77.35	0.0445	0.0003
B^2^	3064.17	0.2559	1	1	104.77	24.37	<0.0001	0.0043
Lack of fit	0	0	4	4				
Total	7640.63	11.98	12	10				

**Figure 9:**
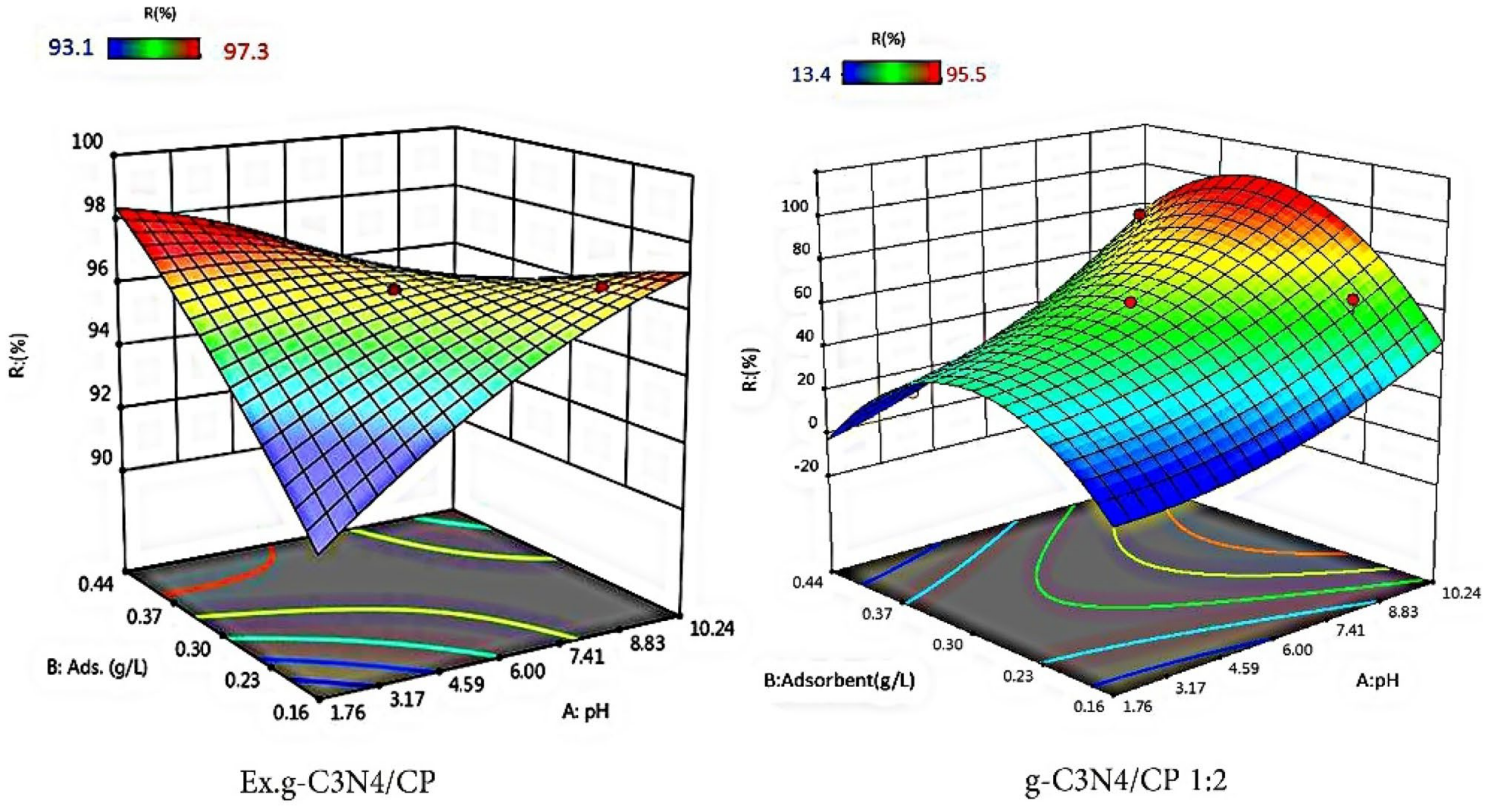
3D response surface: effect of pH versus adsorbent concentration.

The 3D surface response interaction diagram for both composites is presented in Fig. 9. It can be seen that for higher amounts of adsorbent and at alkaline pH, the amount of adsorption has been more efficient. An important parameter in adsorption is pH_pzc_. At this pH, the surface charge of the adsorbent is zero. At pH lower than pH_pzc_, the adsorbent surface has a positive charge and the adsorbent surface has a negative charge at pH higher than pH_pzc_, which tends to adsorb cations. The pH_pzc_ value of g-C_3_N_4_/CP 1:2 and Ex.g-C_3_N_4_/CP adsorbents was determined to be 6.5 and 6.2, respectively. Based on the pH_pzc_ values of the two adsorbents, at pH higher than pH_pzc_, their surface has a negative charge and the electrostatic attraction between the adsorbent and MB as a cationic pollutant with a positive charge increases the amount of adsorption. The increase in adsorption with higher amounts of adsorbent can be attributed to the increase in the adsorbent surface and the availability of more adsorption sites^40^. At pH lower than pH_pzc_, due to the increase of H^+^ ions in the solution, electrostatic repulsion has occurred and less adsorption has occurred.

The optimization of the investigated operating parameters was defined by choosing the minimum amount of adsorbent and the maximum achievable removal efficiency (Table 5).

**Table 5:** Optimum operating conditions for the adsorption of MB (10 ppm).

Variable	The optimal value	
	g-C_3_N_4_/CP1:2	Ex.g-C_3_N_4_/CP
A:pH	9	9
B: Adsorbent concentration (g/L)	0.33	0.2
R (%)	86.3	96.5

In the following, the adsorption kinetics, isotherm, thermodynamic study and regeneration and the stability of the adsorbents in the process in the resulting optimal conditions have been investigated. To further explore the adsorption process, four kinetic models were used in the study (Table 6)^41^. Elovich’s model showed excellent linearity with high fitting, which suggested that the adsorption process might be controlled by chemical adsorption, which is in agreement with the isotherm results.

**Table 6:** Pseudo-first-order, pseudo-second-order, Elovich and intra particle adsorption kinetics for composites.

EX.g-C3N4/CP	g-C3N4/CP 1:2		
0.93	0.93	R^2^	Pseudo first order $\ln \left( {q_{e} - q} \right) = \ln q_{e} - Kt$
0.0261	0.0261	K	
0.96	0.96	R^2^	Pseudo second order $\frac{t}{q} = \frac{1}{{Kq_{e}^{2} }} + \frac{t}{{q_{e} }}$
0.036	0.001	K	
0.97	0.97	R^2^	Elovich $q = \frac{1}{\beta } Ln\left( {\alpha \beta } \right) + \frac{1}{\beta } Ln t$
2.59	2.568	α	
0.048	0.047	β	
0.01	0.86	R^2^	Intra particle $$q_{ } = k_{i} t^{{{\raise0.7ex\hbox{$1$} \!\mathord{\left/ {\vphantom {1 {2 }}}\right.\kern-0pt} \!\lower0.7ex\hbox{${2 }$}}}} + c$$
0.0873	0.8108	*k*_i_	
32.911	18.348	*c*	

To further analyze the adsorption isotherm quantitatively of MB onto the g-C_3_N_4_/CP1:2 and Ex.g-C_3_N_4_/CP, four typical isotherm models were used (Table 7). It could be noted that the equilibrium adsorption capacities increased when the MB concentration was increased. The Langmuir model presumes adsorption occurs as a mono-layer on a homogenous surface. The Freundlich model describes the multiple-layer adsorption on a heterogeneous surface^42, 43^.

**Table 7:** Isotherm of Langmuir, Freundlich, Dubinin Radoshkevich and Temkin by composites.

Ex.g-C_3_N_4_/CP	g-C_3_N_4_/CP 1:2		
0.98	0.95	R^2^	Langmuir $\frac{1}{{q_{e} }} = \frac{1}{{q_{m} }} + \frac{1}{{k_{l} q_{m} }}\frac{1}{{c_{e} }}$
0.008	0.042	R_L_	
54.34	34.12	qm (mg/g)	
2.63	3.959	KL(L/mg)	
0.96	0.69	R^2^	Freundlich $\ln q_{e} = \ln k_{f} + n\ln c_{e}$
0.1277	0.1112	n	
35.05	23.29	Kf[mg/g(mg/l)^−1/n^]	
0.98	0.95	R^2^	Dubinin Radoshkevich $\ln q_{e} = \ln q_{m} - \beta \varepsilon^{2}$
71.5	39.68	q_m_	
1*10^−3^	7*10^−5^	β(mol^2^Kj^−2^)	
70.71	84.515	Ea(Ea = 1/((2β)^0.5^)	
0.97	0.73	R^2^	Temkin $q_{e} = \beta \ln k_{T} + \beta \ln C_{e}$
5.3695	2.7481	(KJ/mol)β	
776.65	6904.9	kT (L/g)	

The value of R^2^ is regarded as the good-to-fit of the experimental data on the Langmuir model. Obviously, the adsorption of MB on g-C_3_N_4_/CP1:2 and Ex.g-C_3_N_4_/CP was better simulated by the Langmuir adsorption isotherm than the others and the values of *q*_*m*_ (34.1 and 54.3 mg/g) calculated by the Langmuir adsorption isotherm basically agreed with the experimental data, which speculated that the adsorption on the composites was monolayer adsorption. Also, such adsorption capacity demonstrated that the Ex.g-C_3_N_4_/CP was more effective adsorbents for MB adsorption than g-C_3_N_4_/CP1:2. For the purpose of evaluating the adsorption feasibility, the separation factor RL was obtained. As we can see from Table 7, the R_L_ values were between 0 and 1, suggesting the MB adsorption was a favorable process^44^.

According to Table 8, the positive values of ∆*H*^*o*^ and the negative ∆*G*^*o*^ values for both composites indicated that the adsorption of MB on them is endothermic and spontaneous. As the temperature rose from 25 to 45 °C, the value of ∆*G*^*o*^ became more negative, leading to the stronger adsorption, which demonstrated that it was in favor of the adsorption for MB at a high temperature. The positive value of ∆*S*^*o*^ indicated an increase in randomness and a significant change in the internal structure of the adsorbent.

**Table 8:** Thermodynamic parameters of MB adsorption on g-C_3_N_4_/CP 1:2 and Ex.g-C_3_N_4_/CP adsorbents.

Temperature (°C)	K		ΔG^0^ (J/mol)		ΔH^0^ (J/mol)		ΔS^0^ (J/mol.k)	
	Ex.g-C_3_N_4_/CP	g-C_3_N_4_/CP 1:2	Ex.g-C_3_N_4_/CP	g-C_3_N_4_/CP 1:2	Ex.g-C_3_N_4_/CP	g-C_3_N_4_/CP 1:2	Ex.g-C_3_N_4_/CP	g-C_3_N_4_/CP 1:2
25	15.18	26.31	− 6689.4	− 8101.66	59,529.07	53,922.94	221.1	207.866
35	36.9	49.89	− 9818.5	− 10,012.38				
45	72.8	103.54	− 11,104.1	− 12267.47				

Table 9 gave the comparison of the adsorption capacity of different sorbents for MB adsorption. It could be seen that the Ex.g-C_3_N_4_/CP possessed similar or higher adsorption capacity of MB compared to the other commonly used adsorbents.

**Table 9:** Comparison of the adsorption capacities for MB on various adsorbents.

Adsorbent	Adsorbent (g/L)	MB (mg/L)	pH	Isotherm	Adsorption capacity (mg/g)	Ref
Fe–AC	0.5–2	50–250	3–9	Langmuir	357.1	^4^
silica polyacrylic acrylamide	1.66	5	6	Langmuir	375.9	^5^
g-C_3_N_4_ nanosheets	0.3	20	7	Langmuir	42.1	^21^
CN-525 CN-550 CN-575	0.1	10	6	Langmuir	37.76 10.32 61.92	^45^
HEC/SiO_2_/C_3_N_4_	0.5	1200	–	Langmuir	132.5	^46^
g-C_3_N_4_/CP 1:2	0.33	10	9	Langmuir	34.1	Current study
Ex.g-C_3_N_4_/CP	0.2	10	9	Langmuir	54.3	

### Regeneration and stability of adsorbents

**Figure 10:**
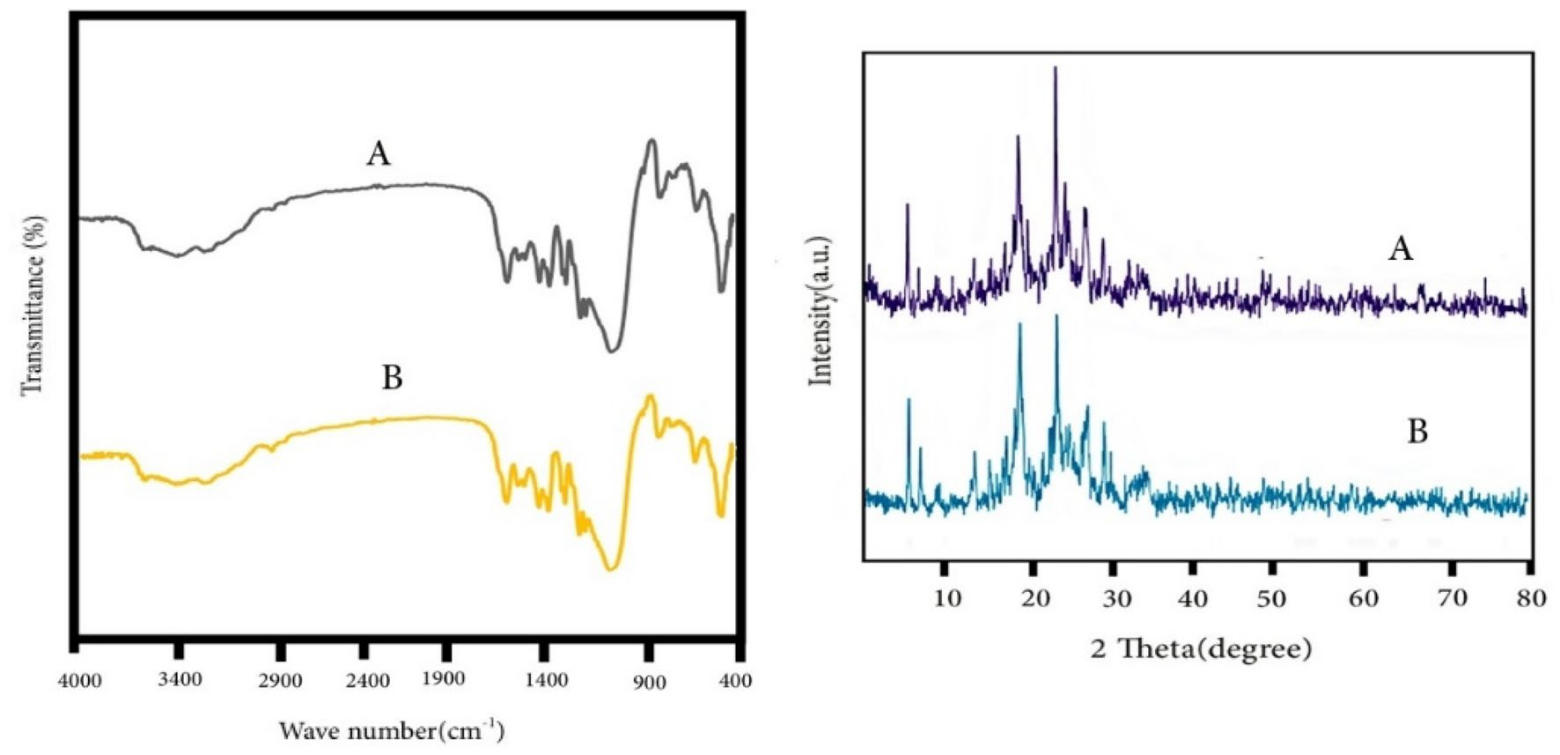
XRD pattern and FT-IR spectrum of Ex.g-C_3_N_4_/CP composite (**A**) before and (**B**) after MB adsorption.

Recyclability of adsorbents was crucial for their practical applications. Thus, in the next step, the regeneration of the g-C_3_N_4_/CP1:2 and Ex.g-C_3_N_4_/CP was elucidated by photocatalytic process. Their reusability (g-C_3_N_4_/CP: 0.33 g/L and Ex. g-C_3_N_4_/CP: 0.2 g/L) in removing MB (10 ppm, pH = 9) was studied for 180 min. After each experiment, the adsorbent was separated, dispersing in water and exposed to visible light radiation (500 watts for 7 h). Then, the adsorbent was separated and used in the next cycle of adsorption. This was repeated three times. The results indicated that both composites have good chemical stability and simple reusability for three cyclic removal of MB (Table 10). Also, the used composite was analyzed by XRD and FT-IR analysis (Fig. 10), which indicates its good stability.

**Table 10:** Investigation of photocatalytic regeneration of g-C_3_N_4_/CP 1:2 and Ex.g-C_3_N_4_/CP adsorbents in three consecutive cycles.

Adsorbent	First cycle	Second cycle	Third cycle
g-C_3_N_4_/CP1:2	86.3	82.3	74.6
Ex.g-C_3_N_4_/CP	96.5	82.3	79.4

## Conclusion

Herein, a novel exfoliated graphitic carbon nitride/Clinoptilolite (Ex.g-C_3_N_4_/CP) with porous structure was successfully prepared via a facile method using urea as the g-C_3_N_4_ precursor. The as-prepared nanocomposite was characterized by XRD, FT-IR, SEM and BET. Batch experiments were carried out under various conditions, such as the amount of adsorbent and solution pH. In optimizing the operational parameters (Adsorbent concentration-pH of solution) by g-C_3_N_4_/CP1:2 and Ex.g-C_3_N_4_/CP composites, the optimal conditions were obtained as 0.33 g/L-pH = 9 and 0.2 g/L-pH = 9, respectively. Ex.g-C_3_N_4_/CP was demonstrated a high adsorption capacity of 54.3 mg.g^−1^, which is much higher than that of single g-C_3_N_4_, or other synthesized composites and many reported adsorbents. The removal efficiency of the as-prepared composite was significantly elevated owing to the synergistic effects. The MB adsorption process on it can be well described using a Langmuir isotherm and Elovich kinetic model. The thermodynamic study suggested that the adsorption of MB was a spontaneous, chemisorption and endothermic process. After the dye adsorption, Ex.g-C_3_N_4_/CP was regenerated simply and retained a high adsorption efficiency for MB after three uses. Our findings were indicated that Ex.g-C_3_N_4_/CP is a low cost, green and promising adsorbent and low environmental impact which can potentially be applied for the removal of extensive pollutants from aqueous solution.

## Data Availability

The datasets used and/or analyzed during the current study available from the corresponding author on reasonable request.

## Kinetic

q_e_: Equilibrium adsorption capacity (mg/g)
q: Adsorption capacity at time t (mg/g)
K: Quasi-first-order adsorption equilibrium constant (1/min)
α: Initial adsorption rate (mg/g.min)
β: Elovich's constant
K_i_: Rate constant of intraparticle penetration (mg.g/min^2^)
C: Intraparticle diffusion constant (mg/g)

## Isotherm

R_L_: Equilibrium parameter
q_m_: Maximum adsorption capacity (mg/g)
K_L_: Langmuir constant
n: Freundlich’s constant, indicating the adsorption intensity
K_f_: Freundlich constant, indicating the adsorption capacity [mg/g (mg/L)^−^^1/n^]
β: Dubinin-Radoshkovich constant related to the average free energy of adsorption (mol^2^Kj^−^^2^)
E_a_: Average free energy of adsorption (E_a_ = 1/((2β)^0.5^)
k_T_: Equilibrium constant corresponding to the maximum bond energy
C_i_: Initial concentration of MB
C_e_: Concentration of MB at the equilibrium
V: Volume of MB solution (L)
M: Mass of the adsorbent (g)

## Thermodynamic

∆*G*^*o*^: Gibbs free energy change (J/mol)
∆*H*^*o*^: Enthalpy change (J/mol)
∆*S*^*o*^: Entropy change (J/mol.K)
*R*: Gas constant (J·mol^−1^·k^−1^)
*T*: Kelvin temperature (K)
*K*: Distribution coefficient (q_e_/C_e_)
